# Disease-linked supertrafficking of a potassium channel

**DOI:** 10.1016/j.jbc.2021.100423

**Published:** 2021-02-16

**Authors:** Hui Huang, Laura M. Chamness, Carlos G. Vanoye, Georg Kuenze, Jens Meiler, Alfred L. George, Jonathan Patrick Schlebach, Charles R. Sanders

**Affiliations:** 1 Department of Biochemistry, Vanderbilt University, Nashville, Tennessee, USA; 2 Center for Structural Biology, Vanderbilt University, Nashville, Tennessee, USA; 3 Department of Chemistry, Indiana University, Bloomington, Indiana, USA; 4 Department of Pharmacology, Northwestern University Feinberg School of Medicine, Chicago, Illinois, USA; 5 Departments of Chemistry and Pharmacology, Vanderbilt University, Nashville, Tennessee, USA; 6 Department of Bioinformatics, Vanderbilt University Medical Center, Nashville, Tennessee, USA; 7 Department of Medicine, Vanderbilt University Medical Center, Nashville, Tennessee, USA

**Keywords:** cardiovascular disease, arrhythmia, ion channel, KCNQ1, mutation, trafficking, AF, atrial fibrillation, cDNA, complementary DNA, CHO, Chinese hamster ovary, ER, endoplasmic reticulum, FC, flow cytometry, FBS, fetal bovine serum, GOF, gain of function, HEK293, human embryonic kidney 293, KCNE, voltage-gated potassium channel-regulatory subfamily E, KCNQ, voltage-gated potassium channel subfamily Q, Lep, leader peptidase, LOF, loss of channel function, LQTS, long QT syndrome, PE, phycoerythrin, VSD, voltage-sensor domain

## Abstract

Gain-of-function (GOF) mutations in the voltage-gated potassium channel subfamily Q member 1 (KCNQ1) can induce cardiac arrhythmia. In this study, it was tested whether any of the known human GOF disease mutations in KCNQ1 act by increasing the amount of KCNQ1 that reaches the cell surface—“supertrafficking.” Seven of the 15 GOF mutants tested were seen to surface traffic more efficiently than the WT channel. Among these, we found that the levels of R231C KCNQ1 in the plasma membrane were fivefold higher than the WT channel. This was shown to arise from the combined effects of enhanced efficiency of translocon-mediated membrane integration of the S4 voltage-sensor helix and from enhanced post-translational folding/trafficking related to the energetic linkage of C231 with the V129 and F166 side chains. Whole-cell electrophysiology recordings confirmed that R231C KCNQ1 in complex with the voltage-gated potassium channel-regulatory subfamily E member 1 not only exhibited constitutive conductance but also revealed that the single-channel activity of this mutant is only 20% that of WT. The GOF phenotype associated with R231C therefore reflects the effects of supertrafficking and constitutive channel activation, which together offset reduced channel activity. These investigations show that membrane protein supertrafficking can contribute to human disease.

The voltage-gated potassium channel subfamily Q member 1 (KCNQ1) (K_V_7.1) plays a critical role in the cardiac action potential (1, 2, 3). Inherited missense mutations in *KCNQ1* encode amino acid changes in the protein that can trigger either loss of channel function (LOF) or aberrant gain of function (GOF) (4, 5, 6, 7). LOF mutations cause type 1 long QT syndrome (LQTS) cardiac arrhythmia, a cause of sudden death. GOF mutations are much less common and cause other arrhythmias such as short QT syndrome and atrial fibrillation (AF). We recently conducted a study on 50 different mutant forms of KCNQ1 in which the single-mutation sites were all located in the voltage-sensor domain (VSD) (8). It was seen that 31 of the mutants exhibited LOF, consistent with their association with LQTS. For a majority of these mutants, reduced cell surface channel levels were observed and documented to result from the combined impact of proteasomal degradation and impaired trafficking to the cell membrane. For the majority of LOF mutants, the most common underlying defect resulting in degradation or mistrafficking was seen to be destabilization of the folded state of the channel. These results provided a well-documented example of what is very likely a general principle regarding disease-promoting LOF mutations in human membrane proteins—that the most common cause of LOF is mutation-induced destabilization of the native conformation (9), leading to misfolding and, often, degradation *via* the endoplasmic reticulum (ER)–associated degradation pathway (10, 11).

The fact that LQTS-linked KCNQ1 LOF is commonly caused by reduced surface channel levels led us to hypothesize that known human disease mutations resulting in aberrant gain of KCNQ1 function might in some cases result from an increase in its cell surface level arising from elevated overall expression, decreased channel degradation, and/or enhanced surface-trafficking efficiency. In this article, we survey the known disease-linked GOF mutant forms of KCNQ1 and report that roughly half do indeed traffic to the cell surface more efficiently than the WT protein. Among these “supertrafficking” mutants, R231C stands out as an extreme example. Follow-up studies of this R231C mutant illuminate the biophysical basis for its supertrafficking trait and provide insight into how this mutation results in a complex disease phenotype in humans.

## Results

### Do KCNQ1 GOF disease mutants supertraffic?

**Figure 1:**
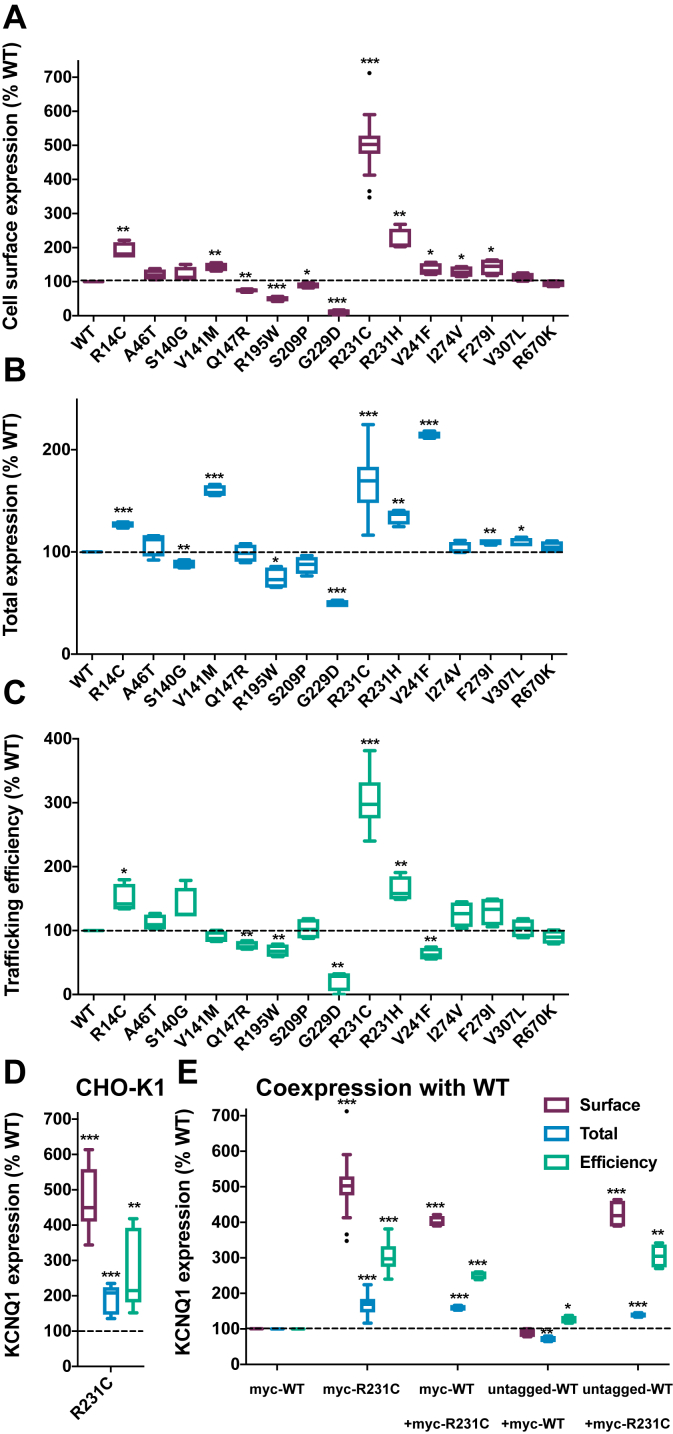
**Total and cell surface expression levels and trafficking efficiencies for disease-linked KCNQ1 gain-of-function mutant forms.** The cell surface expression level (*A*), total cell expression level (*B*), and surface-trafficking efficiency (*C*) of gain-of-function mutants of KCNQ1 were quantitated in transiently transfected HEK293 cells by flow cytometry. The WT trafficking efficiency is 15.4% (±0.7%). *D*, R231C also supertraffics in CHO-K1 cells. *E*, HEK293 cells were transfected with 0.5 μg WT or R231C DNA (homozygous conditions) or cotransfected with 0.25 μg WT and 0.25 μg R231C DNA (heterozygous conditions). Data are shown as percent of WT after correction for nonspecific staining of mock-transfected cells. The results represent 22 experiments for R231C in HEK293 cells, eight experiments for R231C in CHO cells, and four experiments for all other samples. About 2500 single-cell measurements were made per experiment. All panels present the data as Tukey box plots are presented where the central line denotes the median, the box denotes quartiles, and the whiskers denote the values within 1.5 times of the interquartile range. Outliers are plotted as individual points (*cf.*, R231C data in panel *A*). ∗*p* < 0.05, ∗∗*p* < 0.01, and ∗∗∗*p* < 0.001 for comparison of the mean value of each mutant with the corresponding WT value by Student's *t* test. CHO, Chinese hamster ovary; HEK293, human embryonic kidney 293 cells; KCNQ1, voltage-gated potassium channel subfamily Q member 1.

We first examined the expression of 15 previously identified disease mutations reported to cause GOF in KCNQ1 (12, 13, 14, 15, 16, 17, 18, 19, 20, 21, 22, 23, 24, 25, 26, 27, 28, 29, 30, 31, 32), as summarized in Table S1. GOF mutations were introduced into complementary DNA (cDNA) encoding KCNQ1 bearing a myc epitope in an extracellular loop connecting the S1 and S2 transmembrane segments, a modification that has no impact on channel folding, trafficking, or function (33). Each GOF variant was then transiently expressed in human embryonic kidney 293 (HEK293) cells, and the expression of each variant was measured by flow cytometry–based quantification of anti-myc immunostaining. Two distinct fluorescently tagged anti-myc antibodies were used to differentially label cell surface and intracellular KCNQ1 levels (8). From these measurements, it was possible to calculate the cell surface–trafficking efficiency, which is defined as [(level of KCNQ1 at surface)/(total cellular KCNQ1)] × 100. Results are shown in Figure 1. Panel 1*A* shows that GOF mutant forms of KCNQ1 exhibit a wide range of surface expression levels relative to WT, ranging from about 10% for G229D to 500% for R231C. Seven of the 15 GOF mutants were seen to traffic more efficiently than the WT KCNQ1. Of these, R231C stood out because of its fivefold more efficient trafficking to the plasma membrane relative to WT. R231C KCNQ1 is a supertrafficker. Because it represents an extreme example, we focused all our subsequent work on investigating R231C. The fivefold increase in surface expression of R231C reflects the combined effects of its 1.7-fold higher level of total expression relative to WT (Fig. 1*B*) and its threefold greater surface-trafficking efficiency (Fig. 1*C*).

For R231C, the trafficking results shown in Figure 1 were not dependent on the expression system. Similar results were obtained when Chinese hamster ovary (CHO)-K1 cells (Fig. 1*D*) were used for channel expression instead of HEK293 cells. We also examined whether the coexpression of R231C affected the WT channel surface trafficking. When myc-R231C was cotransfected with myc-WT (same amount of total myc-R231C + myc-WT cDNA as myc-R231C cDNA in the previous experiments), the cell surface–trafficked myc-tagged protein is fourfold higher than when the myc-tagged WT channel is expressed alone (Fig. 1*E*). This indicates that coassembled WT/R231C KCNQ1 heterotetramers also supertraffic.

**Figure 2:**
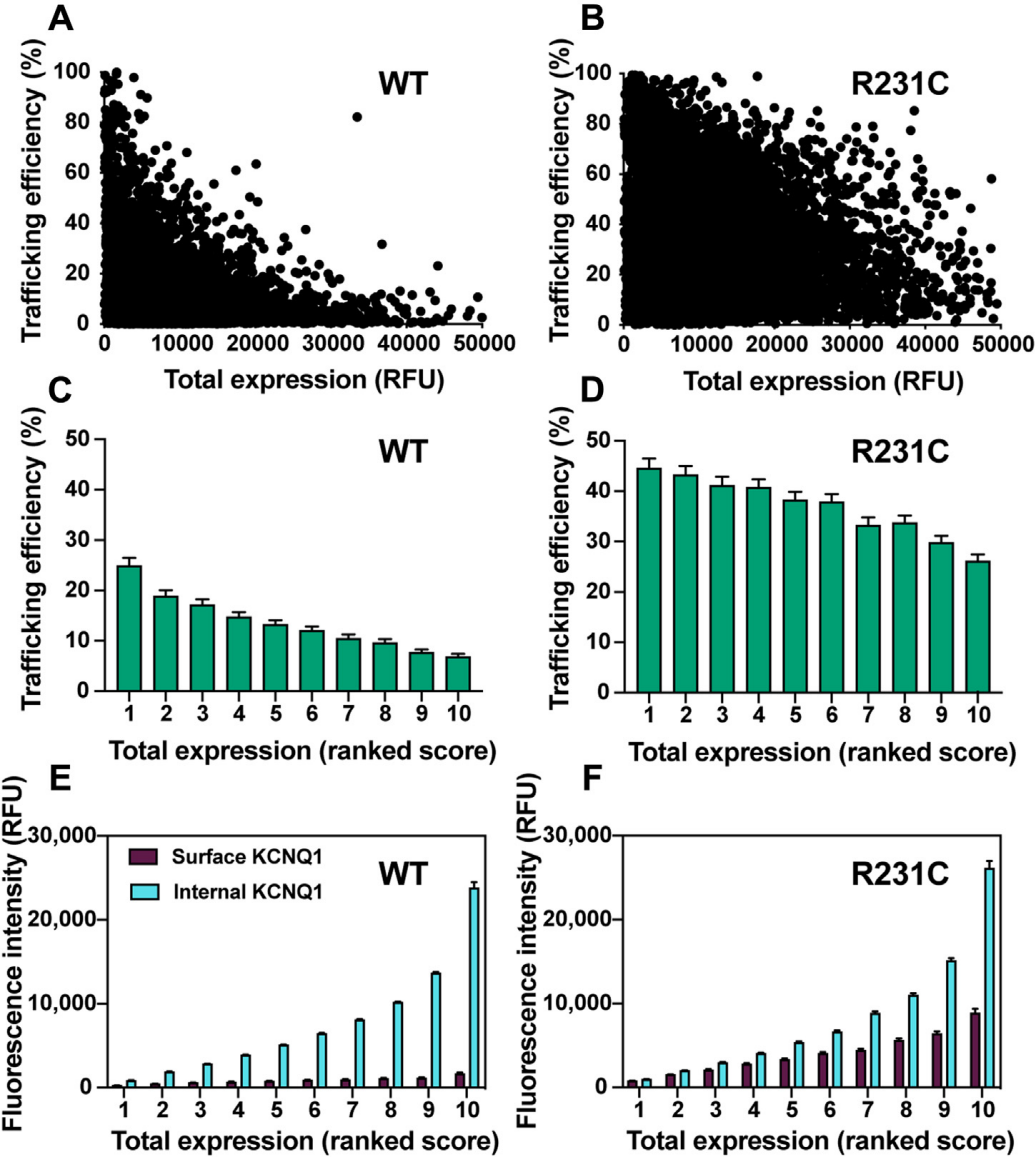
**Single-cell trafficking properties are analyzed for WT *versus* R231C KCNQ1 in HEK293 cells.** The internal expression and trafficking efficiency of WT and R231C were determined for each of the 7500 cells from the three independent biological replicates. Cells that exhibited a KCNQ1 trafficking efficiency of zero were excluded from this analysis. *A* and *B*, plots of single-cell KCNQ1 trafficking efficiency, where trafficking efficiency = (KCNQ1 surface expression level for each cell/KCNQ1 total expression for each cell) × 100. Results were ordered from lowest to highest KCNQ1 total expression and then binned into ten groups, designated 1 (for the 10% of cells exhibiting the lowest total KCNQ1 expression levels) to 10 (for the 10% with the highest total KCNQ1 expression levels). The KCNQ1 trafficking efficiency (*C* and *D*) and surface *versus* internally trapped levels (*E* and *F*) have been plotted as a function of the ten bins ranked based on the single-cell total expression levels. Panels *C* and *D* are a replot of the data shown in panels *A* and *B*, respectively. The bar height is the mean value, and the error bar shows the 95% confidence interval for each group; KCNQ1, voltage-gated potassium channel subfamily Q member 1.

Figure 2, *A* and *B* shows the single-cell trafficking efficiencies for WT and R231C expressing cells as a function of total cellular expression. The average trafficking efficiency of WT KCNQ1 is 15 ± 1% (SEM), whereas the trafficking efficiency of R231C is much higher with an average trafficking efficiency of 43 ± 2%. When these data were binned into ten equally sized cohorts (ranked by total expression in single cells), the single-cell trafficking efficiency of both WT and R231C KCNQ1 was seen to be inversely proportional to total single-cell expression (Fig. 2*C*). However, while R231C trafficking efficiency does decline with higher expression levels, the drop is not as steep as for WT (Fig. 2*D*). Figure 2, *E* and *F* provides further clarification by showing that as the total expression of both WT and R231C increase, the levels of both surface-trafficked and cell-internal KCNQ1 increase, but that for WT, the internally trapped fraction grows more steeply. This suggests that R231C undergoes forward trafficking through the protein-folding quality control system of the ER more readily than the WT channel.

### Supertrafficking of R231C KCNQ1 is unrelated to disulfide bond formation

**Figure 3:**
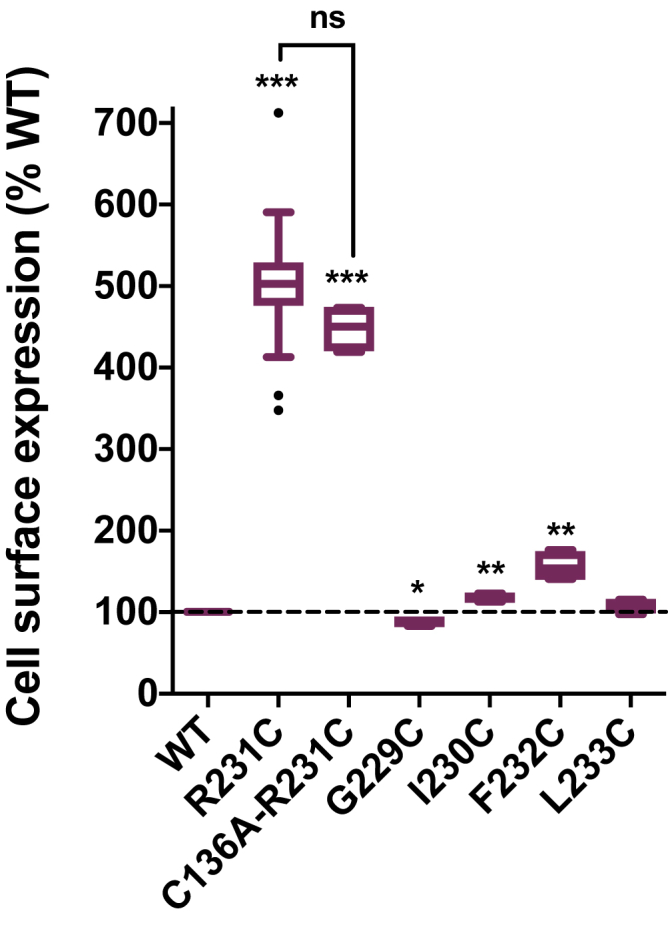
**Supertrafficking of R231C KCNQ1 is unrelated to disulfide bond formation.** Residue R231 is proximal to C136 in S1 when the channel is in the intermediate state, indicating that C231 could conceivably form a disulfide bond with C136. An Ala mutation was introduced to avoid any such potential disulfide bond. Residues surrounding R231 have been mutated to Cys to investigate whether mutation of any of the site 231-proximal positions leads to supertrafficking. See the legend to Figure 1 for additional details. ∗*p* < 0.05, ∗∗*p* < 0.01, and ∗∗∗*p* < 0.001 for comparison of the mean value of each mutant with the corresponding WT value by Student's *t* test. KCNQ1, voltage-gated potassium channel subfamily Q member 1; ns, not significant for comparison of the mean value of indicated two mutants.

We considered the possibility that supertrafficking could be related to the disulfide bond formation between the introduced Cys231 in S4 and the only native cysteine residue in its general vicinity, C136, located in the S1 transmembrane helix. As shown in Figure 3, the C136A/R231C double mutant exhibits elevated cell surface levels similar to R231C, ruling out intramolecular disulfide bond formation as a causative or contributing factor to supertrafficking of this mutant. We also examined the consequences of mutating G229, I230, F232, or L233 to cysteine (instead of R231). None of these KCNQ1 mutants exhibited supertrafficking (Fig. 3), indicating that the supertrafficking effect of mutation to cysteine is specific to R231C.

### Mutations of R231 to other amino acids lead to a range of KCNQ1 trafficking efficiencies

**Figure 4:**
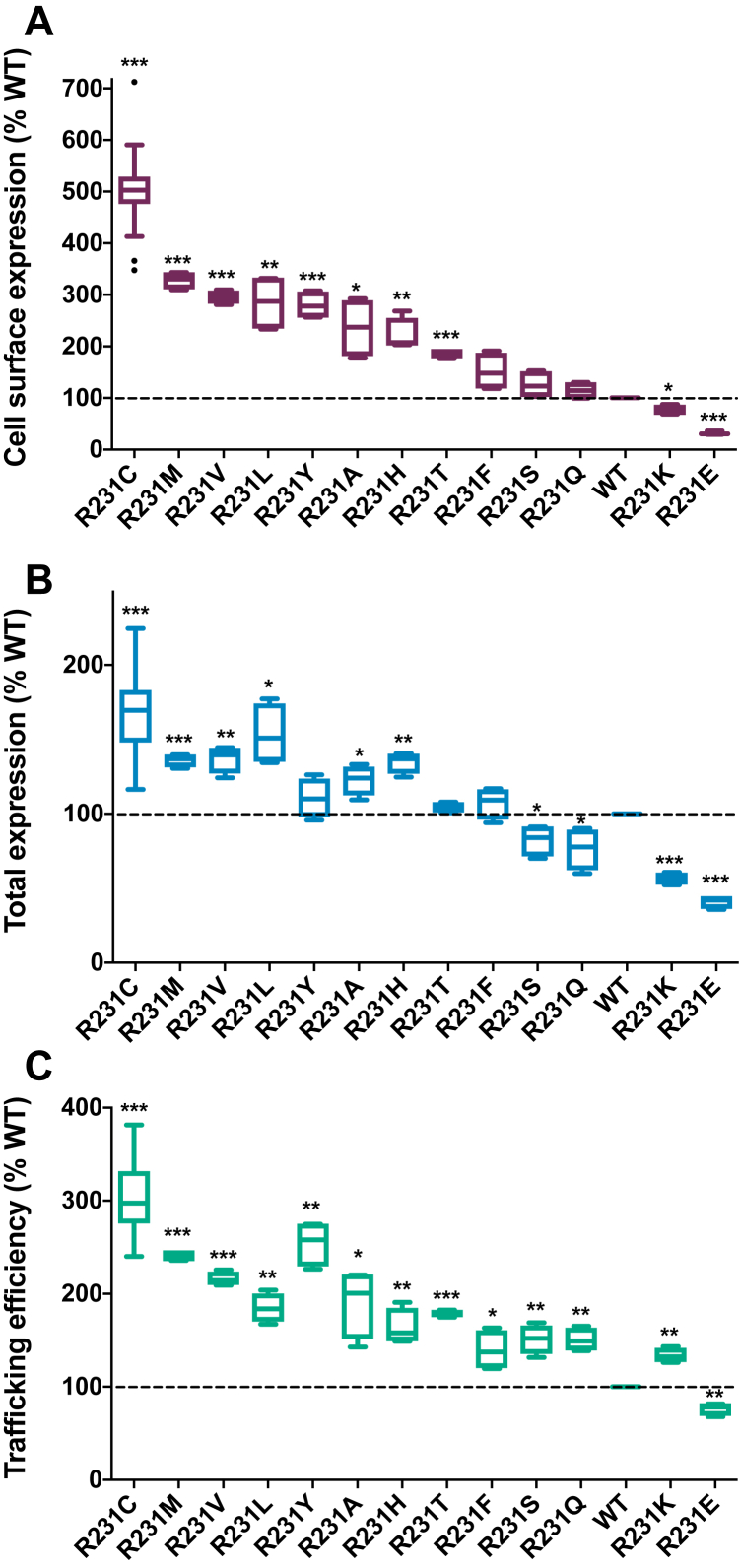
**Mutations of R231 to other amino acids lead to a range of KCNQ1 trafficking efficiencies in HEK293 cells.***A,* The cell surface levels of the KCNQ1 R231 mutants. *B,* The total (surface + internal) levels. *C,* The trafficking efficiencies. See the legend to Figure 1 for additional details. ∗*p* < 0.05, ∗∗*p* < 0.01, and ∗∗∗*p* < 0.001 for comparison of the mean value of each mutant with the corresponding WT value by Student's *t* test. HEK, human embryonic kidney; KCNQ1, voltage-gated potassium channel subfamily Q member 1.

We next quantitated the expression and trafficking of a series of 13 R231 mutants (Fig. 4). R231C exhibits the highest level of expression as well as the highest surface expression and trafficking efficiency. However, other mutants exhibit supertrafficking to a lesser degree, with trafficking efficiencies on the order of twofold to threefold higher than the WT being observed for R231M, R231V, R231L, and R231Y. Conversely, substitution of R231 to more polar residues (lysine and glutamate) results in lower-than-WT total and surface expression levels.

### Supertrafficking of R231 mutants is related to the membrane integration of the KCNQ1 S4 helix

**Figure 5:**
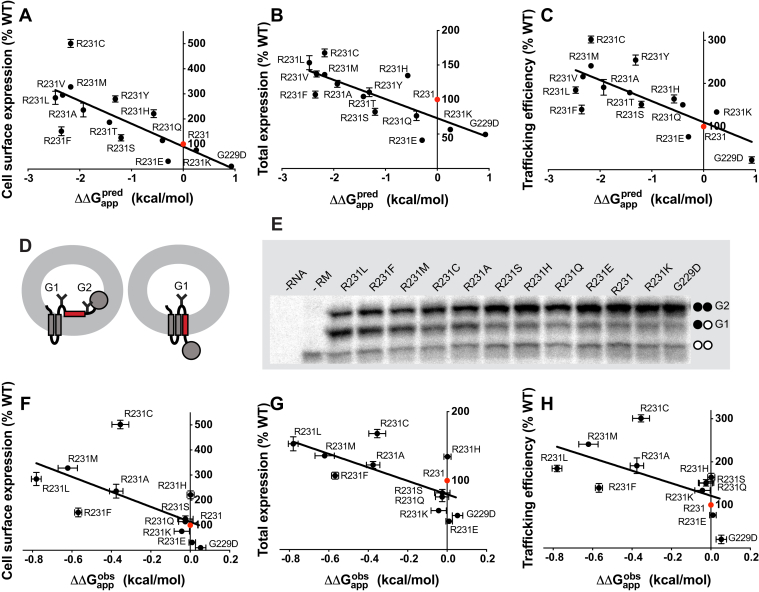
**The supertrafficking of the R231C mutant is related to the increased membrane integration of the KCNQ1 S4 helix.***A*–*C*, the Hessa/White/von Heijne biological hydrophobicity scale helps to explain the trafficking of some 13 different KCNQ1 site 231 mutants in HEK293 cells. Here, $\Delta \Delta G_{app}^{pred}$ is the predicted (http://dgpred.cbr.su.se/) relative to WT for the apparent-free energy difference between correct integration of the site 231-containing KCNQ1 S4 segment into ER membrane by the Sec61 translocon *versus* the fraction that is predicted not to integrate into the membrane. *D*–*H*, *in vitro* translation results for site 231 mutants of the isolated KCNQ1 S4 segment show a generally excellent correlation with the expression of the same mutant series in full-length KCNQ1 expressed in HEK293 cells. The assay used a Lep construct for which its H-segment was replaced with the KCNQ1 S4 segment (*red*). Chimeric Lep constructs encoding KCNQ1 mutants were transcribed and translated *in vitro* with canine rough microsomes (RMs) and analyzed by SDS-PAGE. Doubly (*G2*) and singly (*G1*) glycosylated Lep proteins were quantified. Unglycosylated products are labeled with two *white dots*. Control reactions were performed without adding RNAs or RMs. $\text{ Δ}G_{app}^{obs}$ values were determined from three (R231M) or four (all other mutants) independent biological replicates and compared with that for WT to generate the reported $\text{ΔΔ}G_{app}^{obs}$. Data plotted are the mean values, and the error bar is SEM. ER, endoplasmic reticulum; HEK, human embryonic kidney; KCNQ1, voltage-gated potassium channel subfamily Q member 1; Lep, leader peptidase.

R231 is located within the S4 helix, a key element of the VSD of KCNQ1 and other voltage-gated ion channels. The S4 helix contains positively charged residues (Arg or Lys), which are uncharacteristic for a transmembrane helix (34, 35, 36). While these Arg and Lys residues are critical to the voltage-sensing function of S4, they potentially compromise the cotranslational membrane integration of this helix into the membrane of the ER (34, 37, 38, 39, 40). In the context of the full-length KCNQ1 channel, this would likely result in misfolding of the channel and failure to surface traffic. This is consistent with our observation that WT KCNQ1 traffics to the membrane surface with only roughly 15% efficiency (Fig. 2*A*). We hypothesized that substituting R231 with more hydrophobic residues would enhance membrane integration of S4 into the membrane of the ER, which in turn would increase the folding and trafficking efficiency of the channel. We first examined this issue by comparing the trafficking results of Figure 4 to the projected effects of these mutations on the membrane integration of S4 as predicted by the Hessa/White/Von Heijne “biological hydrophobicity scale,” a knowledge-based energy scale for the translocon-mediated membrane integration of nascent transmembrane helices (41, 42). Figure 5 shows that the surface level, total expression, and surface-trafficking efficiency of R231 variants are highly correlated with the predicted effects of these mutations on the transfer-free energy of the S4 helix. Overall, trafficking of the 13 mutants examined in this work appears to be closely related to mutation impact on the hydrophobicity of S4. However, as shown in Figure 5, *A*–*C*, R231C is an outlier that expresses and traffics at even higher levels than would be expected based on the trend for the other mutants. Indeed, its surface expression level is roughly twofold higher than expected based on the best linear fit of the site 231 mutant series expression levels to the Hessa/White/von Heijne predicted ΔΔG values.

Based on these observations, it is unclear whether the deviation in expression caused by the R231C mutation stems from its effects on the membrane integration of the S4 helix, its impact on the structural properties of the full-length channel, or some combination of the two. To examine the effects of the R231C mutation on the topological preferences of S4, we experimentally measured the membrane integration of S4 variants bearing mutations at residue 231 in the context of a chimeric leader peptidase (Lep)/S4 helix fusion protein (41). These Lep/S4 fusion proteins were expressed *via in vitro* translation in canine rough microsomes, and the membrane integration efficiency of each S4 variant was inferred from the relative abundance of the singly glycosylated Lep (indicating correct membrane integration of S4) and doubly glycosylated Lep (indicating aberrant lumenal translocation of S4) (Fig. 5, *D* and *E*). Consistent with observations for other ion channel S4 segments (38, 40), the WT S4 segment of KCNQ1 exhibits a marginal propensity to partition into the membrane (ΔG_app_ = 0.56 ± 0.03 kcal/mol; Fig. S1A and Fig. 5*E*). Measured ΔG_app_ values plateau around a value of ∼0.6 kcal/mol for variants bearing non-native charges at residue 231, and the experimentally measured change in the apparent free energy of membrane integration relative to WT (ΔΔG_app_) caused by these mutations is small relative to the predicted values (Fig. S1). This deviation between experiment and prediction for the polar substitutions likely reflects the limited dynamic range of this assay (38, 43). Nevertheless, the introduction of hydrophobic side chains at R231 clearly increases the efficiency of S4 membrane integration and decreases ΔG_app_, as expected (Fig. 5*E*). With the exception of R231C, measured ΔΔG_app_ values are inversely correlated with the surface expression, total expression, and trafficking efficiency of full-length KCNQ1 mutants (Fig. 5, *F*–*H*). While this general correlation suggests that the KCNQ1 expression is highly sensitive to the topological energetics of S4, the observed deviation of R231C implies that the change in the membrane integration efficiency cannot fully account for its elevated surface trafficking—there must be at least one additional mechanism that contributes to its surface trafficking.

### Energetic coupling of the R231C, F166, and V129 side chains is a second factor that contributes to the supertrafficking of R231C KCNQ1

We next sought to determine if interactions between the R231C side chain and those of proximal residues in the folded conformation of the KCNQ1 VSD contribute to channel supertrafficking. To identify the structural interactions that influence KCNQ1 trafficking, we quantitated the surface expression of a series of single and double mutants that selectively perturb possible contacts. Our initial strategy was based on the notion that surface expression should be negatively attenuated by single mutations that disrupt a critical interaction but restored or even enhanced by the introduction of the reciprocal (swapped sites) mutation. A positive result in such studies indicates what we refer to as “energetic linkage” of the two sites in a way that promotes folding and surface trafficking. Observation of any such energetic linkage may reflect not only direct physical action of the two sites but also an indirect interaction mediated through an allosteric network.

**Figure 6:**
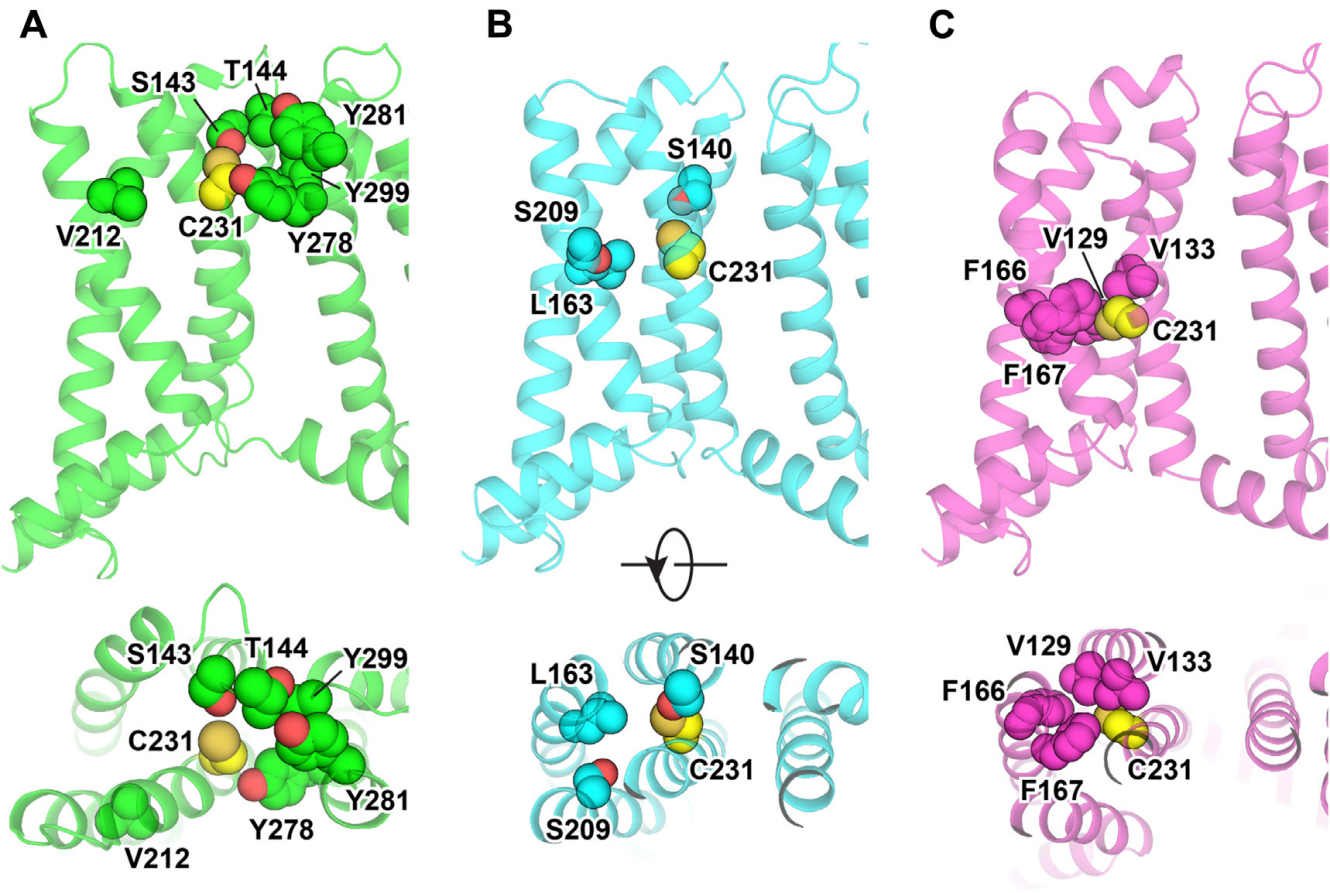
**Locations of residues used for double-mutant analysis of inter-residue side chain interactions involving C231 are illustrated in KCNQ1 structures.** Residues that are proximal to position 231 in current experimental and model structures of the activated, intermediate, and resting states of KCNQ1 are shown. *A*, human KCNQ1 activated state structure (Protein Data Bank: 6V01) (44). *B*, Rosetta model of the KCNQ1 intermediate state structure developed based on the experimental KCNQ1 voltage-sensor domain NMR structure (Protein Data Bank: 6MIE) (45). *C*, Rosetta model of the KCNQ1 resting state (46). The native R231 in these structural models was substituted for Cys, and the models were minimized with Rosetta. The side chains of C231 and its proximal residues are depicted as *spheres*. KCNQ1, voltage-gated potassium channel subfamily Q member 1.

**Figure 7:**
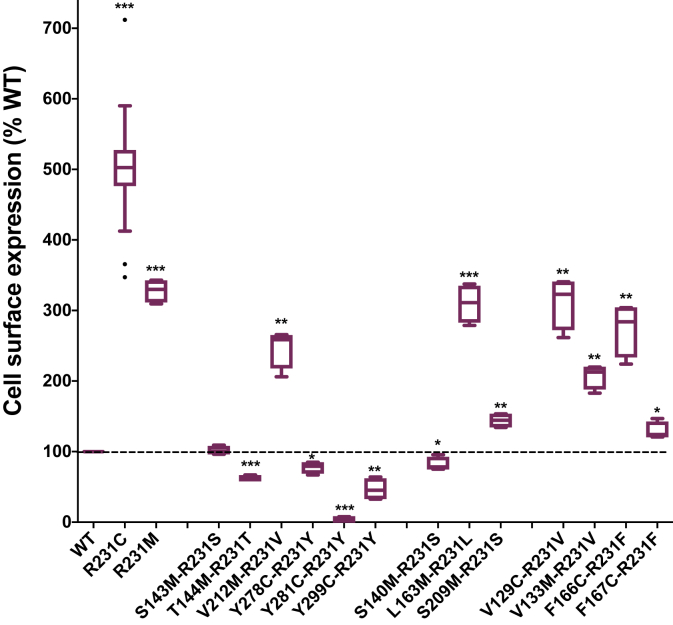
**Swapped site double mutagenesis scan of sites that are possibly in direct contact with KCNQ1 site 231 based on available structures of the protein to identify pairs of site for which interaction may impact surface trafficking.** Thirteen residues that are proximal to residue 231 in at least one functional state have been identified from current KCNQ1 structures, including S143, T144, V212, Y278, Y281, and Y299 in the activated state, S140, L163, and S209 in the intermediate state, and V129, V133, F166, and F167 in the resting state. See the legend to Figure 1 for additional details. ∗*p* < 0.05, ∗∗*p* < 0.01, and ∗∗∗*p* < 0.001 for comparison of the significance of the mean value for each mutant relative to the corresponding WT value by Student's *t* test. KCNQ1, voltage-gated potassium channel subfamily Q member 1.

We selected potential interacting sites with R231C using the available experimental structures for the fully activated human KCNQ1 channel (44) and intermediate-activated VSD (45), as well as a recently developed inactive state model (46). Based on these structures, C231 is predicted to be close to or in contact with sites 143, 144, 212, 278, 281, and 299 in the fully activated state, 140, 163, and 209 in the intermediate state, and 129, 133, 166, and 167 in the inactive state (Fig. 6). To eliminate possible disulfide bond formation involving R231C, we sometimes used R231M (which also supertraffics) as the parent mutant, with key results then being validated by repeating measurements using R231C. Figure 7 shows that the reciprocal mutations of the side chain of R231C or R231M with the WT side chains of the following residues led to complete or near-complete loss of supertrafficking: S140, S143, T144, F167, S209, Y278, Y281, and Y299. While it is possible that these side chains may interact with residue 231 in certain conformational states of the channel, these results suggest that any such interaction does not contribute to the supertrafficking trait of the R231C channel. This inference was definitively confirmed by additional mutagenesis measurements involving these sites, as shown in Fig. S2.

**Figure 8:**
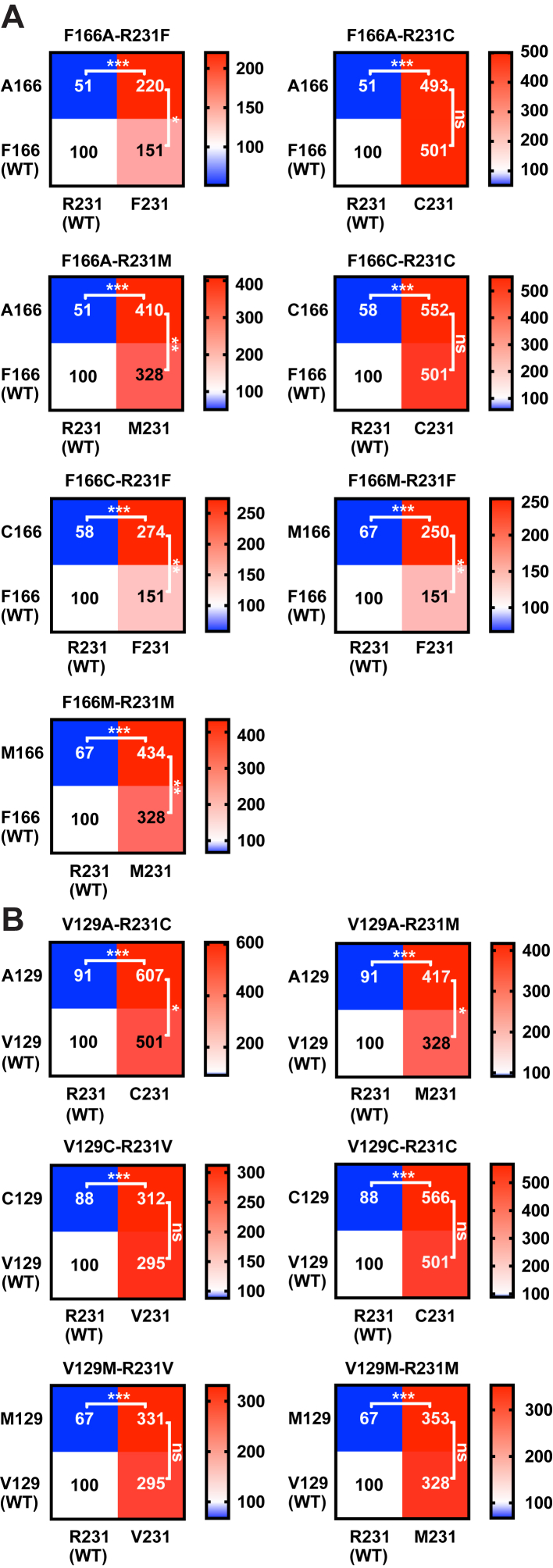
**Double-mutant cycle experiments to probe possible coupling interaction of KCNQ1 sites 166 and 129 with site 231.***A*, KCNQ1 site 166 and *B*, KCNQ1 site 129. *Box plots* of these same data are described for Fig. S3. Here, each 2 × 2 grid plot presents the double-mutant cycle analysis for the mutations from WT to the double mutant indicated above in each grid plot. Each grid presents the surface expression levels of WT KCNQ1 (*lower left box* in each plot), a site 231 mutant (*lower right box*), a site 166 or 129 mutant (*upper left box*), and the corresponding site 231 plus site (166 or site 129) double mutant (*upper right box*). Each *x*-axis gives the amino acid at site 231, and each *y*-axis gives the amino acid at site 166 or 129. Each box includes the measured surface level of the channel both as a number and in a heat map form (see *color key* to the right of each plot), in both cases where the surface-trafficked level of KCNQ1 is percent relative to WT. The statistical significance of the difference between the surface level of each double mutant relative to the parent single-site mutants is in each case given next to the tie lines: ∗*p* < 0.05, ∗∗*p* < 0.01, and ∗∗∗*p* < 0.001 for comparison of the mean value of each double mutant with the corresponding parent mutant by Student's *t* test. ns, not significantly different in comparison with the corresponding parent mutant; KCNQ1, voltage-gated potassium channel subfamily Q member 1.

Figure 7 also shows several possible R231C-interactor sites that retain considerable supertrafficking capability when mutationally swapped with site 231 Cys or Met: V129, V133, L163, F166, and V212. Follow-up double-mutant cycle analysis studies were conducted to explore the possibility of trafficking—impacting energetic linkage between the side chains of these sites and that of site 231 (Fig. 8 and Figs. S2 and S3). For sites V133, L163, and V212, the additional mutagenesis data (Fig. S2) do not confirm the significant energetic linkage: nonconservative mutation of each of these sites in the presence of R231C or R231M resulted in either no impact or a negative change in surface trafficking that was in no case rescued by a subsequent swap of that residue with Cys231 or Met231. Instead, the impact of mutations at these sites on trafficking is independent and additive with the impact of the mutations at site 231. In contrast, for F166 and V129, the data shown in Figure 8 (Fig. S3) are consistent with a trafficking—impacting energetic linkage between their side chains and that of either R231C or R231M. Figure 8*A* shows that the mutation of F166 to Ala, Cys, or Met in WT (R231) KCNQ1 results in reduced trafficking relative to WT. However, when the mutation of F166 to any of these amino acids is combined with the mutation of R231 to Cys, Met, or Phe, each double mutant (Fig. 8*A*) supertraffics. Indeed, for five of the seven double mutants, surface trafficking is enhanced even beyond what is seen for the parent R231 mutant, with the two exceptions still supertrafficking at a level comparable to the corresponding parent R231 mutant. For example, the F166M mutant traffics to the surface with reduced efficiency (67%) relative to WT. However, the F166M/R231M surface traffics to an even higher degree (434%) than the R231M mutant (328%). Sites R231C and F166 are evidently energetically coupled in a way that increases the trafficking efficiency. A similar pattern is seen for V129 (Fig. 8*B*), where V129A traffics less efficiently than WT, but the combination of V129A with either the R231C or the R231M mutations leads to an enhancement of the supertrafficking for the double mutants (Fig. 8*B*). These results indicate that site 129 is also energetically coupled with R231C.

**Figure 9:**
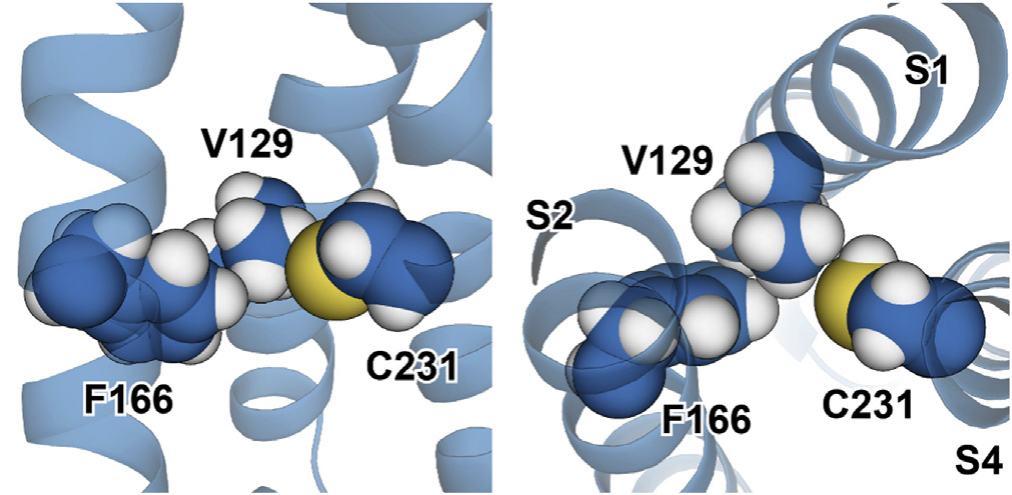
**Tripartite interaction of the energetically coupled side chains of C231, V129, and F166 in the inactive state structure** (46) **of the KCNQ1 voltage-sensor domain.** It is seen that while C231 does not directly contact F166, V129 forms an intimate bridge between C231 and F166. Both side (*left*) and (*right*) top views are shown. KCNQ1, voltage-gated potassium channel subfamily Q member 1.

From the available 3D structures of the KCNQ1 VSD, we see that the side chain of R231C is close to the side chains of V129 and F166 only when the VSD populates the inactive state (Figs. 6 and 9). When R231 is computationally mutated to cysteine within the inactive state structure, its side chain interacts directly with V129, whereas the side chain of V129 is in intimate direct contact with F166 (Fig. 9). Based on these observations, coupling between R231C site and V129 site is likely direct, whereas coupling between R231C and F166 is likely mediated by V129. Modeling also suggests that it is possible that the longer site 231 side chains of WT R231 or R231M could interact directly with both V129 and F166.

### Channel function and activation defects in R231 variants

**Figure 10:**
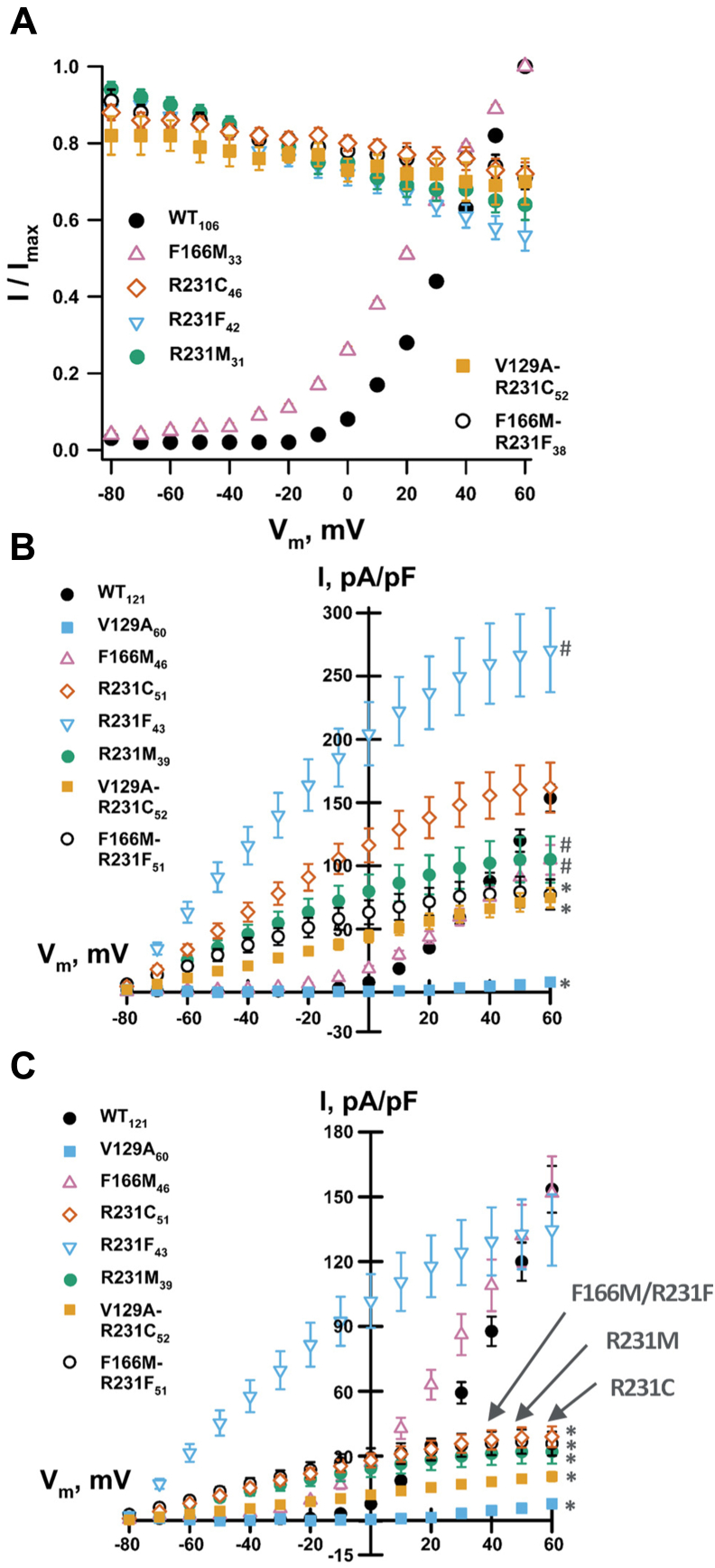
**Electrophysiological functional analysis validates energetic coupling between residues 166 and 231 and shows that R231C and related mutants are not only constitutively active but also have reduced activity relative to the WT channel.** CHO-K1 cells stably expressing KCNE1 were electroporated with plasmids containing either WT or mutant KCNQ1 cDNA. *A*, voltage dependence of the activation curves obtained from the whole-cell currents recorded from CHO_KCNE1 cells transiently expressing WT or mutant KCNQ1 channels. Results for V129A are not shown because the tail currents were very small and could not be fitted. *B*, current density–voltage relationships measured in CHO_KCNE1 cells transiently expressing WT or mutant KCNQ1 channels. *C*, current density–voltage relationships measured in CHO_KCNE1 cells transiently expressing WT or mutant KCNQ1 channels and normalized based on the measured quantity of channel protein at the CHO cell plasma membrane (Table S2). Whole-cell currents were recorded using automated patch clamp, normalized by membrane capacitance, and the number of recorded cells (n) is labeled next to each mutant. #*p* ≤ 0.02 and ∗*p* ≤ 0.001 for all pairwise comparison of the mean value current measured at +60 mV of each mutant and corresponding WT value by one-way ANOVA. cDNA, complementary DNA; CHO, Chinese hamster ovary; KCNE1, voltage-gated potassium channel regulatory subfamily E member 1; KCNQ1, voltage-gated potassium channel subfamily Q member 1.

WT and selected supertrafficking KCNQ1 channel mutants were functionally analyzed using a CHO-K1 cell line stably expressing potassium voltage-gated channel subfamily E member 1 (KCNE1). KCNE1 is a channel accessory subunit that pairs with KCNQ1 to form the channel complex responsible for the delayed rectifier I_Ks_ current of the cardiac action potential (47). Figure 10*A* shows that the supertrafficking KCNQ1 mutants R231C, R231F, R231M, F166M/R231F, and V129A/R231C exhibited constitutive channel activity in the −80 to +60 mV voltage range. The results for these R231 mutants are distinct from those for WT and F166M KCNQ1 (Fig. 10*A*), which open only upon membrane depolarization, with a V_1/2_ of roughly +30 mV.

Figure 10*B* depicts the average current–voltage relationships measured after pulses of 2 s from CHO_KCNE1 cells transiently transfected with WT, V129A, F166M, R231C, R231M, R231F, V129A–R231C, or F166M–R231F KCNQ1. The whole-cell current density measured at +60 mV for R231C is similar to that for WT, whereas R231F exhibits significantly larger currents. R231M, F166M, V129A–R231C, and F166M–R231F exhibit lower current density than the WT channel at +60 mV. Even though the V129A mutant traffics similarly to WT (Fig. 8*B* and Table S2), it exhibited very low current (Fig. 10*B*) and can be regarded as an LOF mutant.

Figure 10*C* replots the current density measured for each mutant KCNQ1 channel following normalization of the data to the cell surface expression level as measured in CHO-K1 cells (Table S2). The normalized currents measured at +60 mV reveal that the R231C and R231M KCNQ1 channels exhibit decreased single-channel function relative to WT (Fig. 10*C*). While these mutants are constitutively open, their relative activity per channel at the membrane is only on the order of 20% that of WT. In contrast, the R231F and F166M mutants exhibit similar activity at +60 mV when compared with the WT channel (*p* = 0.862 when comparing both mutants). The activities of the F166M–R231F and V129A–R231C double mutants are only ca. 20 and 15% of WT, respectively (*p* ≤ 0.001; Fig. 10*C*). These results suggest that energetic coupling seen between certain pairs of side chains at sites 166 and 231 (Fig. 8*A*) can contribute not only to supertrafficking but also to a significant reduction in channel function by decreasing channel conductance, lowering open state probability, or both. We again noted that sites 166 and 231 appear to be in proximity (bridged by contact with V129; Fig. 9) only when the channel populates the inactive state. This suggests that the decrease in channel activity seen for R231C and R231M is due to the reduced open state probability. These mutations likely stabilize the nonconductive inactive state, suggesting an equilibrium between the states for R231C and R231M mutant channels in which the inactive-to-active state population ratio is roughly 4:1 in favor of the inactive state. It is interesting that the R231F mutant, which just barely supertraffics (150% of WT; Fig. 4), exhibits single-channel activity at 60 mV that is similar to WT (Fig. 10*C*). Unlike R231C and R231M, R231F evidently continues to favor the active state conformation.

Overall, the results shown in Figure 10 are consistent with previous observations (13, 20, 48) that certain mutations of R231 to neutral residues result in constitutive (voltage-independent) channel activation. However, these results also advance our understanding by showing that some of these very same mutations, including R231C, exhibit significantly reduced channel activity relative to WT. Moreover, the observation that the side chain of R231C is energetically coupled with V129 and F166 in a way that is coupled with channel trafficking is consistent with the proximity of the side chains of these residues, which occurs only in the inactive state. This leads to a working model for how R231C results in disease-associated KCNQ1 dysfunction, as described later.

## Discussion

### Supertrafficking of membrane proteins as a potential disease mechanism

Among the 15 known disease-linked GOF KCNQ1 mutations, seven traffic more efficiently than the WT channel, indicating that supertrafficking is not a particularly rare phenomenon among GOF KCNQ1 mutants. These results establish a paradigm for supertrafficking as a mechanism that can sometimes contribute to human diseases linked to mutant membrane proteins. In this regard, we noted that one of the neuronal paralogs of KCNQ1, KCNQ2, is known to be subject to GOF mutations that cause a severe disease, early infantile epileptic encephalopathy. One of these GOF mutants is R201C, which occurs at the VSD site corresponding to the KCNQ1–R231 (49). We hypothesize that the mechanisms of early infantile epileptic encephalopathy–causative GOF associated with KCNQ2–R201C likely includes supertrafficking. An additional possible example is provided by another neuronal paralog, KCNQ3, for which the corresponding R230C mutation was recently identified as a genetic risk factor for autism spectrum disorder (50, 51). This mutant has not yet been tested to determine if it exhibits GOF and supertrafficking traits. Finally, we point to the voltage-gated SCN5A sodium channel as another protein for which supertrafficking is likely to be a contributing disease mechanism. There are more than 60 GOF mutant forms of SCN5A that cause LQTS type 3, including voltage-sensor R → C mutations (7). Evidently, only additional data will determine whether this hypothesis is correct for the other channels.

### Supertrafficking of the R231C KCNQ1 channel offsets the reduced single-channel activity of this mutant

Dominant missense mutations in the human *KCNQ1* can result in the formation of defective channel proteins. While a majority of the known KCNQ1 disease variants exhibit channel LOF that results in LQTS arrhythmia, a smaller set of variants result in what is thought to be aberrant GOF, resulting in short QT syndrome or familial AF (4, 5, 6, 7) (Table S1). The R231C mutant has previously been included in this classification because it is known to be constitutively conductive at all transmembrane potentials (13, 20, 48, 52), as confirmed in this work. This is in contrast to healthy conditions where gating of the KCNQ1 channel in complex with its modulatory subunit KCNE1 is voltage regulated. The results of this work revealed two additional traits that set the R231C mutant apart from WT and some other GOF mutant forms. One is that while it is constitutively active, the single-channel activity of R231C in complex with KCNE1 seems to be only 20% that of the WT KCNQ1/KCNE1 complex. The other is that this mutant is much more abundant at the plasma membrane than the WT channel (Fig. 1), an observation that holds even under R231C/WT heterozygous conditions (Fig. 1*E*). This increase in channel levels at the plasma membrane offsets the mutation-induced decrease in channel activity. Accordingly, arrhythmia caused by the R231C KCNQ1 appears to be due to supertrafficking of the channel as compounded by voltage-independent channel activity. Together, these two GOF mechanisms offset the reduced channel activity. Our results provide clarity regarding how R231C KCNQ1 results in pleiotropic arrhythmic phenotypes in human patients including AF, LQTS, and fetal bradycardia (13, 20, 52, 53). We suggest that depending on exactly in which part of the heart the channel is expressed, the delicate balance between supertrafficking and dysregulation of channel activity (GOF effects) *versus* reduced channel functionality (LOF) is likely to vary, such that R231C-like I_Ks_ may locally exhibit either net LOF properties (promoting LQTS) or GOF behavior (promoting superimposed AF).

### Why does R231C supertraffic?

We observed that the mutation of R231 to hydrophobic residues generally increases the abundance of KCNQ1 at the cell surface, with the results for R231C being especially striking. The supertrafficking activity of this mutant reflects the combined effects of its ∼1.7-fold higher-than-WT total level of expression and its threefold higher surface-trafficking efficiency. Our results suggest that these effects arise from two contributing factors. First, about half of the supertrafficking can be attributed to enhancement by this mutation of the efficiency of correct translocon-mediated membrane integration of the S4 helix during KCNQ1 biogenesis (Fig. 5). Second, the WT R231 residue contributes to the polarity of the S4 transmembrane segment, resulting in inefficient membrane integration of WT KCNQ1 and an enhanced propensity to misfold within the ER. Despite this, R231 is highly conserved because of its indispensable role in transmembrane voltage sensing. How nature has dealt with the functional imperative of placing cationic residues in transmembrane VSD sites has previously been discussed (34, 38, 40). Given that action potentials hinge on a precise sequence of controlled ionic fluxes, the native expression levels of WT KCNQ1 have very likely been carefully tuned by evolution so as to account for the fact that a WT S4 is functionally essential but does not integrate into the membrane with 100% efficiency, even under healthy conditions. These findings for KCNQ1 parallel recent investigations of the cotranslational folding of rhodopsin, where it was found that its expression and trafficking were highly sensitive to mutations that alter the hydrophobicity of a polar transmembrane domain containing functionally constrained polar residues (54, 55).

In addition to the effect of the R231C mutation on the efficiency of cotranslational folding of KCNQ1, we found that this mutation enhanced the trafficking to the cell surface of the channel in a way that is linked to the energetic coupling of the R231C thiol with the V129 and F166 side chains. Enhancement by the R231C mutation of this three-residue interaction appears to shift the voltage sensor equilibrium to favor the inactive state relative to the activated state by roughly 4:1, leading to the reduced conductance seen for the R231C channel. This C231/V129/F166 tripartite interaction (Fig. 9) evidently also results in the extra twofold increase in surface trafficking beyond that based on the enhanced membrane integration of S4 that occurs when R231 is replaced by the more hydrophobic cysteine. The simplest interpretation is that stabilization of the inactive state VSD conformation at a transmembrane potential of 0 V (before it reaches the polarized plasma membrane) relative to the intermediate and fully activated VSD conformations somehow enables the KCNQ1 channel to more efficiently evade the intracellular retention and/or degradation mechanisms associated with protein-folding quality control in the early secretory pathway. However, we cannot rule out the possibility that the R231C mutation could impact KCNQ1 structure or dynamics in additional trafficking—impacting ways that were not revealed by this study.

## Conclusions

Nearly half of the known GOF KCNQ1 mutations traffic more efficiently than WT. This suggests that supertrafficking should be considered as a potential contributing GOF disease mechanism for other channels, such as KCNQ2 and KCNQ3, and possibly for other membrane proteins. This work also illuminates the structural biophysical basis for the extreme supertrafficking exhibited by R231C KCNQ1 and how this trait is both reinforced and offset by other functional defects, resulting in the complex disease phenotype manifest in human carriers of this mutation.

## Experimental procedures

### Plasmids and mutagenesis

The untagged and c-myc–tagged human KCNQ1 DNA (GenBank accession number: AF000571) were engineered in the mammalian expression vector pIRES2-enhanced GFP as previously described (8). The c-myc tag (EQKLISEEDL) was inserted into the extracellular S1–S2 linker between residues Glu146 and Gln147. Human KCNE1 (L28168) was subcloned into the pcDNA3.1(+) vector for biochemical experiments and into pcDNA5/FRT to generate the CHO_KCNE1 cell line used for the functional analysis of KCNQ1 mutants as previously described (8, 56). In-Fusion HD Cloning (Takara Bio) was used to introduce the sequence of the WT S4 domain of KCNQ1 in place of the H-segment of a modified Lep gene, as previously described (42). Mutations in KCNQ1 were introduced by QuikChange site-directed mutagenesis using untagged or c-myc–tagged KCNQ1 plasmid as the template. Mutations in the S4 segment of Lep were generated using the WT S4 Lep as the template. The coding regions of all constructs were verified by Sanger sequencing.

### Cell culture

HEK293 cells (CRL-1573) and CHO-K1 cells (CRL-9618) were purchased from American Type Culture Collection. HEK293 cells were grown in Dulbecco's modified Eagle's medium supplemented with 10% fetal bovine serum (FBS), 10 mM Hepes, 100 units/ml penicillin, and 100 μg/ml streptomycin at 37 °C in 5% CO_2_. CHO-K1 cells were cultured in F-12 nutrient mixture medium supplemented with 10% FBS, 50 units/ml penicillin, 50 μg/ml streptomycin, and 2 mM l-glutamine at 37 °C in 5% CO_2_. As previously described (48), CHO-K1 cells stably expressing KCNE1 (CHO_KCNE1) were made using KCNE1 in pcDNA5/FRT vector and generated using the Flp-In System (Thermo Fisher Scientific) following the manufacturer's instruction. CHO_KCNE1 cells were maintained under selection with hygromycin B (600 μg/ml).

### Flow cytometry

Unless otherwise stated, HEK293 cells were used for the flow cytometry study of KCNQ1 total and surface expression levels. Cells were plated into 6-well plates, and the next day, transiently transfected with 0.5 μg WT or mutant myc-KCNQ1, each well at 30 to 50% confluence. Transfection was performed using Fugene 6 transfection reagent (Promega). When WT and mutant myc-KCNQ1 were cotransfected, the ratio of DNA used was 1:1 (0.25:0.25 μg). When KCNE1 in pcDNA3.1(+) vector and myc-KCNQ1 were cotransfected, the ratio of DNA used was also 1:1 (0.5:0.5 μg).

Approximately, 48 h after transfection, cells were prepared for flow cytometry quantitation. As previously described (8, 57), cells were prepared using the Fix & Perm cell fixation and cell permeabilization kit (Thermo Fisher Scientific) following the supplier's protocol. All procedures were performed at room temperature. Briefly, cells were washed once with PBS flow cytometry (FC) buffer containing 25 mM Hepes and 0.1% sodium azide (PBS–FC; pH 7.4), detached in PBS–FC buffer containing 0.5 mM EDTA and 0.5% bovine serum albumin, and centrifuged at 300*g* for 5 min. Cells were then resuspended in 100 μl PBS–FC containing 5% FBS with 1:100 diluted phycoerythrin (PE)-conjugated anti-myc (9B11) mouse monoclonal antibody (catalog no. 3739; Cell Signaling Technology) and incubated for 30 min in the dark. 100 μl fixation medium was then added to each sample followed by incubation for 15 min to fix cells. Cells were washed once with PBS–FC containing 5% FBS. Cells were then resuspended in 100 μl permeabilization medium with 1:100 diluted Alexa Fluor 647–conjugated anti-myc (9B11) mouse monoclonal antibody (catalog no. 2233; Cell Signaling Technology) and incubated for 30 min in the dark. Cells were washed once, and fluorescence intensities were quantitated using a four-laser Fortessa flow cytometer (BD Bioscience). To correct the fluorescence intensity difference of the two antibodies, cells expressing WT myc-KCNQ1 were fixed, permeabilized, and immunostained with either PE- or Alexa Fluor 647–conjugated anti-myc antibody, and the intensity ratio of the two antibodies was calculated.

Gating on GFP-positive cells was applied to select cells successfully transfected with the KCNQ1 plasmid. Fluorescence of mock-transfected cells was used for background correction. Unless otherwise stated, the expression and trafficking efficiency of mutants were shown as percent of WT, where trafficking efficiency was defined as:

[(surface)_mutant_/(total)_mutant_]/[(surface)_WT_/(total)_WT_] × 100. The mean and SEM values were calculated from at least three independent biological replicates. Data were analyzed and plotted using Prism 8.2 software (GraphPad). Tukey box plot was used to illustrate data distribution, and Student's *t* test was used to compare the differences between WT and mutants by unpaired two-tailed *t* test. A *p* value ≤0.05 was considered to be statistically significant.

### *In vitro* translation of Lep variants

A glycosylation-based biochemical assay was used to measure the cotranslational membrane integration efficiency of S4 variants. The WT and a series of Lep variants bearing mutations of interest within the KCNQ1 S4 segment were generated as described previously. mRNA was synthesized from these plasmids using a RiboMAX SP6 RNA production kit (Promega). *In vitro* translation of the mRNA was carried out using rabbit reticulocyte lysate (Promega), supplemented with canine rough microsomes (tRNA probes) and EasyTag ^35^S-labeled methionine (PerkinElmer) for 1 h at 30 °C. The products were separated on a 12% SDS-PAGE gel. The gel was dried for 1 h at 60 °C, then exposed overnight on a phosphor imaging plate (GE Healthcare) and imaged on a Typhoon Imager (GE Healthcare). Doubly (*G2*) and singly (*G1*) glycosylated Lep proteins were quantified by densitometry in ImageJ software (National Institutes of Health). Apparent transfer-free energy values were calculated according to the following equation:

$$\text{Δ}G_{app}\;=-RT\text{ ln}\left(\frac{G1}{G2}\right)=-RT\;\ln\left(K_{app}\right)$$

where Δ*G*_*app*_ is the apparent transfer-free energy for the transfer of the S4 helix from the translocon to the membrane, *R* is the universal gas constant, *T* is the temperature, *G2* is the amount of doubly glycosylated protein, *G1* is the amount of singly glycosylated protein, and *K*_*app*_ is the apparent equilibrium constant, as previously described (41). Δ*G*_*app*_ is the average of at least three independent biological replicates and compared with the Δ*G*_*app*_ for WT to generate ΔΔ*G*_*app*_ values.

### Electrophysiology experiments

WT and mutant untagged KCNQ1 were transiently transfected into CHO_KCNE1 cells using the Maxcyte STX system as previously described (48). Briefly, cells grown from 70 to 80% confluency were harvested using 5% trypsin. Cells were collected by gentle centrifugation (160*g*, 4 min), followed by washing the cell pellet with 5 ml electroporation buffer (EBR100; MaxCyte Inc) and resuspension in electroporation buffer at a density of 108 viable cells/milliliter. For each electroporation, plasmid encoding KCNQ1 variant (10 μg) was added to 100 μl cell suspension. The DNA–cell suspension mix was then transferred to an OC-100 processing assembly (MaxCyte Inc) and electroporated using the CHO–PE preset protocol. Electroporated cells were grown for 48 h at 37 °C in 5% CO_2_. Following incubation, cells were harvested, counted, transfection efficiency determined by flow cytometry, and then frozen in 1 ml aliquots at 1.5 × 10^6^ viable cells/milliliter in liquid nitrogen until used in experiments.

Electroporated cells were thawed the day before an experiment, plated, and incubated for 10 h at 37 °C in 5% CO_2_.

The cells were then transferred to 28 °C in 5% CO_2_ and grown overnight. Prior to the experiment, cells were passaged using 5% trypsin in cell culture media. Cell aliquots (500 μl) were used to determine the cell number and viability by automated cell counting. Cells were then diluted to 200,000 cells/ml with external solution (see later) and allowed to recover in 40 min at 15 °C while shaking on a rotating platform at 200 rpm.

Currents were recorded at a room temperature in the whole-cell configuration by an automated planar patch clamp using a SyncroPatch 768 PE (Nanion Technologies). Single-hole and 384-well recording chips with medium resistance were used in this study. The external solution (millimolar) contained the following: NaCl 140, KCl 4, CaCl_2_ 2, MgCl_2_ 1, Hepes 10, and glucose 5, with the final pH adjusted to 7.4 with NaOH. The internal solution (millimolar) contained the following: KF 60, KCl 50, NaCl 10, Hepes 10, EGTA 10, and ATP-K2 2, with the final pH adjusted to 7.2 with KOH. Whole-cell currents were not leak subtracted. The contribution of background currents was determined by recording before and after addition of the I_Ks_ blocker JNJ-303 (4 μM). Only JNJ-303–sensitive currents were used for analysis.

Pulse generation and data collection were carried out with PatchController384 V.1.3.0 and DataController384 V1.2.1 software (Nanion Technologies). Whole-cell currents were filtered at 3 kHz and acquired at 10 kHz. The access resistance and apparent membrane capacitance were estimated using built-in protocols. Whole-cell currents were recorded from −80 to +60 mV (10 mV steps) 1990 ms after the start of the voltage pulse from a holding potential of −80 mV. The voltage dependence of activation was calculated by fitting the normalized G–V curves with a Boltzmann function (tail currents measured at −30 mV). The number of cells (n) is given in the legends to the figures. Statistical analysis was performed with one-way ANOVA, and *p* ≤ 0.02 was considered significant.

## Data availability

All data needed to evaluate the conclusions in this article and supporting information are presented in this article or in the supporting information. Correspondence and requests for materials should be addressed to C. R. S. (chuck.sanders@vanderbilt.edu).

## Supporting information

This article contains supporting information (12, 13, 14, 15, 16, 17, 18, 19, 20, 21, 22, 23, 24, 25, 26, 27, 28, 29, 30, 31, 32, 48).

## Conflict of interest

The authors declare that they have no conflicts of interest with the contents of this article.
