# Supplementary material for: Disease-linked supertrafficking of a potassium channel
Source: J Biol Chem. 2021 Feb 16;296:100423. doi: 10.1016/j.jbc.2021.100423 (PMC7988323; doi:10.1016/j.jbc.2021.100423)
Supplement: Supplemental Figures S1–S3 and Tables S1–S2 [file mmc1.pdf]

Supporting Information for:

Disease-linked super-trafficking of a potassium channel

Hui Huang[a,b], Laura M. Chamness[c],

Carlos G. Vanoye[d], Georg Kuenze[b,e], Jens Meiler[b,e,f], Alfred L. George Jr. [d],

Jonathan P. Schlebach[d], & Charles R. Sanders[a,b,g,*]

[1]Department of Biochemistry, Vanderbilt University, Nashville, TN 37240, USA

[2]Center for Structural Biology, Vanderbilt University, Nashville, TN 37240, USA

[3]Department of Chemistry, Indiana University, Bloomington, IN 47405 USA

[4]Department of Pharmacology, Northwestern University Feinberg School of Medicine, Chicago, IL, 60611, USA

[5]Departments of Chemistry and Pharmacology, Vanderbilt University, Nashville, TN 37240, USA

[6]Department of Bioinformatics, Vanderbilt University Medical Center, Nashville, TN 37232, USA

[7]Department of Medicine, Vanderbilt University Medical Center, Nashville, TN 37232, USA

*Correspondence should be addressed to C.R.S, chuck.sanders@vanderbilt.edu

This PDF file includes:

Figure S1
Figure S2
Figure S3
Table S1
Table S2

Figure S1

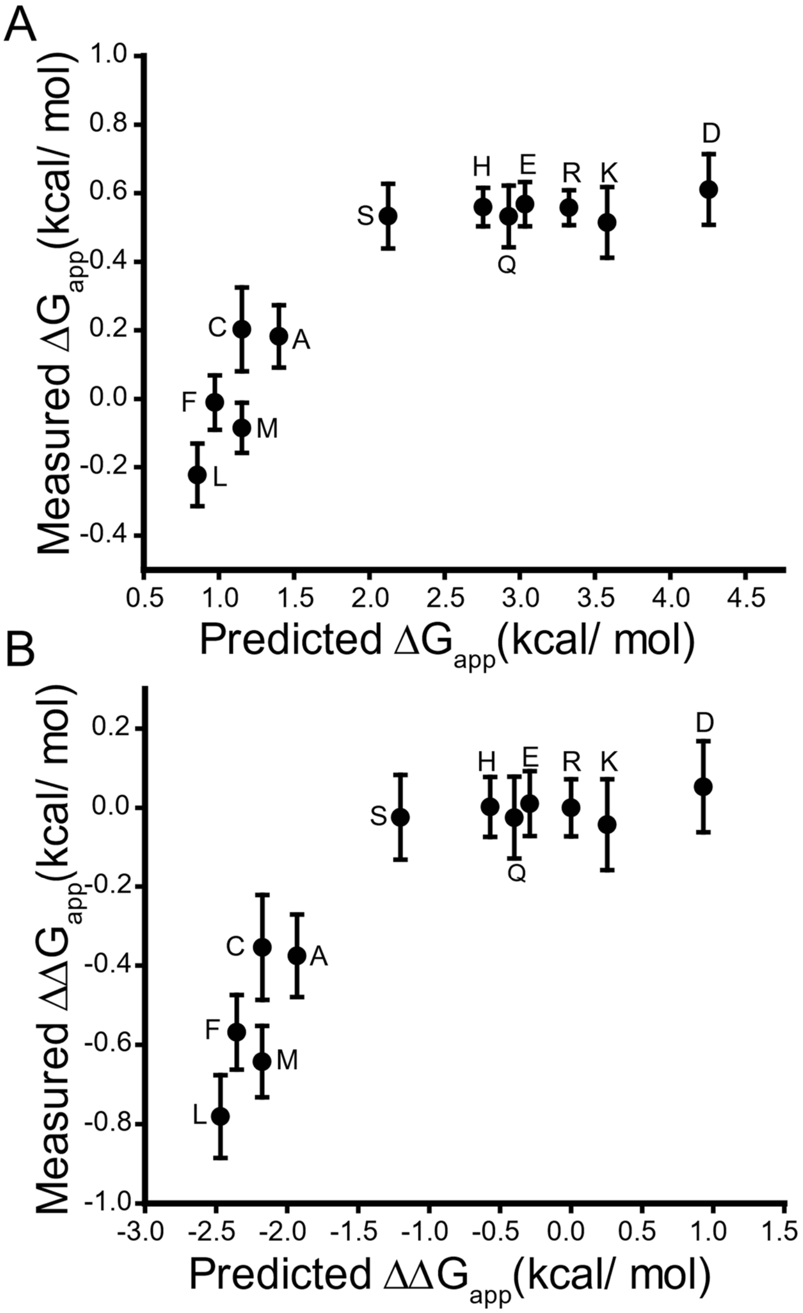

Figure S1. **Predicted and measured apparent transfer free energies for S4 variants. A)** Apparent transfer free energies derived from the glycosylation state of chimeric leader peptidase proteins bearing variants of the S4 helix are plotted against the corresponding predicted transfer free energies generated by the DG predictor (http://dgpred.cbr.su.se/). Error bars reflect the standard deviation from three experimental replicates. The side chain at residue 231 is indicated for reference.

Measured ΔG values plateau above ~0.6 kcal/ mol, which reflects the limited dynamic range of this assay. **B)** The measured change in the apparent transfer free energies derived from the glycosylation state of chimeric leader peptidase proteins bearing variants of the S4 helix are plotted against the corresponding predicted change in transfer free energies generated by the DG predictor (http://dgpred.cbr.su.se/). Error bars reflect the propagated standard deviations from the mutant and WT measurements. The side chain at residue 231 is indicated for reference.

Figure S2

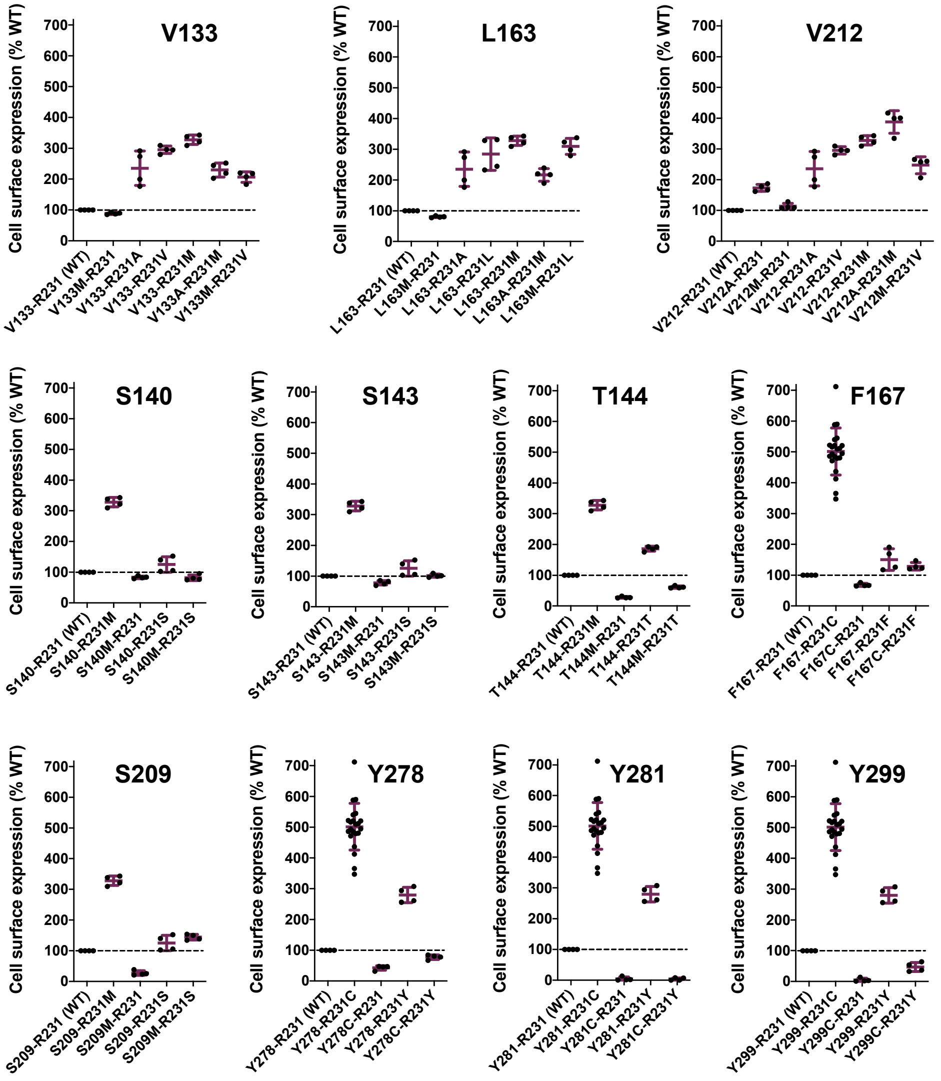

Figure S2. Double mutant analysis results for sites that were deemed not to be energetically coupled to the side chain for site 231 (see Fig. 7). The cell surface expression levels of double mutants and corresponding single mutants have been plotted. Data are expressed as means ± SD of at least four independent biological replicates.

Figure S3

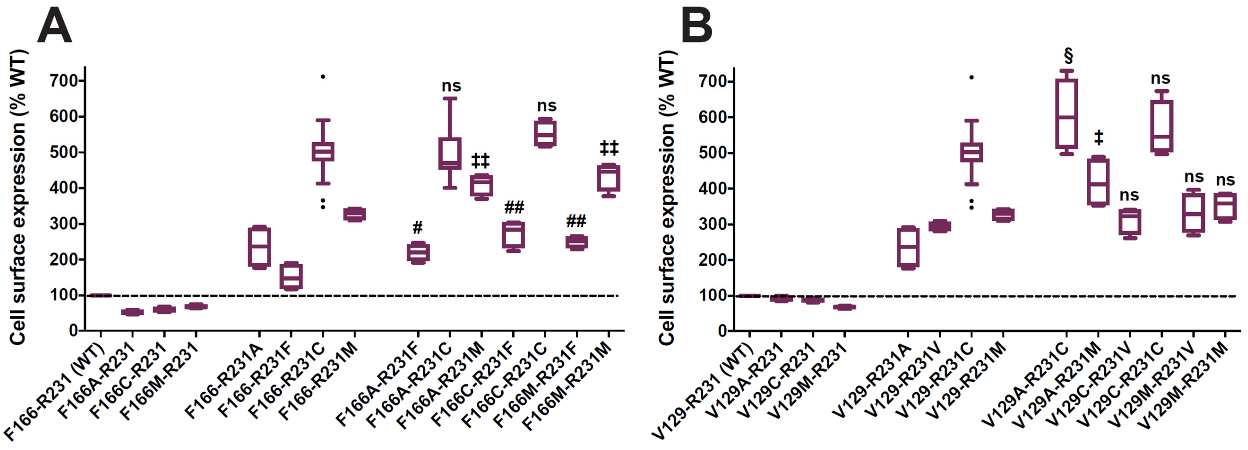

Figure S3. Box plots for the surface trafficking measurements used for double mutant analysis to assess possible energetic coupling of KCNQ1 site 231 with sites 129 and 166. This supporting figure is a re-plot of the data presented in Figure 8 of the main paper. The surface expression levels are presented as percentage relative to WT. **(A)** Series for mutations at sites 231 and 166. **(B)** Series for mutations at sites 231 and 129. #, $P < 0.05$; ##, $P < 0.01$ for comparison of the mean value for the double mutant with the parent R231F mutant. §, $P < 0.05$ for comparison with the parent R231C mutant. ‡, $P < 0.05$; ‡‡, $P < 0.01$ for comparison with the parent R231M mutant. ns: not significant for comparison with the corresponding parent R231 mutants. Comparisons of surface levels of all the double mutants with corresponding parent F166 mutants (F166A, F166C, or F166M) or V129 mutants (V129A, V129C, or V129M) are significant and not labeled in the figure.

Table S1. Properties of disease-linked GOF mutant forms of KCNQ1

Protein Change	Codon Change	Location	Associated Phenotype	ClinVar Interpretation	Functional Property	Reference
**R14C**	CGC>TGC	N-terminus	AF	not provided	w/ E1: WT like; if cell swelling, I_{max} increased, activation $\Delta V_{1/2}$ left shift, slow deactivation.	(28)
**A46T**	GCG>ACG	N-terminus	LQTS, AF	uncertain significance	w/o and w/ E1: I_{max} increased or WT like, fast activation, activation $\Delta V_{1/2}$ WT like.	(26,30,31)
**S140G**	AGC>GGC	S1	AF	pathogenic	w/o E1: fast activation, slow deactivation.<br>w/ E1 or E2: I_{max} drastically increased; activation $\Delta V_{1/2}$ left shift and instantaneous activation, slow deactivation.<br>w/ E3: WT like.	(15,16)
**V141M**	GTG>ATG	S1	SQTS, AF	pathogenic or likely pathogenic	w/o E1: WT like.<br>w/ E1: I_{max} increased, activation $\Delta V_{1/2}$ left shift and instantaneous activation, slow deactivation.	(15,21)
**Q147R**	CAG>CGG	S1-S2 linker	LQTS, AF	not provided	w/ E1: I_{max} reduced, activation $\Delta V_{1/2}$ WT like.<br>w/ E2: I_{max} increased, activation $\Delta V_{1/2}$ WT like.<br>w/o E1, w/ E3 or E4: WT like.	(24)
**R195W**	CGG>TGG	S2-S3 linker	LQTS, AF	uncertain significance	w/o and w/ E1: I_{max} increased; fast activation and slow deactivation.	(22,27,30)
**S209P**	TCC>CCC	S3	AF	pathogenic	w/o E1: slow deactivation.<br>w/ E1: activation $\Delta V_{1/2}$ left shift and instantaneous activation, fast activation, slow deactivation.	(17)
**G229D**	GGC>GAC	S4	LQTS, AF	pathogenic	w/ E1: activation $\Delta V_{1/2}$ left shift and instantaneous activation, I_{max} reduced, slow deactivation.	(19,32)
**R231C**	CGC>TGC	S4	LQTS, AF	pathogenic	w/o E1: slow deactivation.<br>w/ E1: activation $\Delta V_{1/2}$ left shift and instantaneous activation, I_{max} reduced.	(13,20,48)
**R231H**	CGC>CAC	S4	LQTS, AF	pathogenic	w/ E1: activation $\Delta V_{1/2}$ left shift and instantaneous activation.<br>w/ E3: I_{max} reduced.	(12,26)
**V241F**	GTC>TTC	S4	LQTS, AF	uncertain significance	w/ E1: activation $\Delta V_{1/2}$ left shift and instantaneous activation, slow deactivation.	(23)
**I274V**	ATC>GTC	S5	LQTS, SQTS, AF	conflicting interpretations	w/o E1: WT like.<br>w/ E1: I_{max} increased, fast activation, slow deactivation.	(29)
**F279I**	TTT>ATT	S5	SQTS	pathogenic	w/o E: I_{max} increased, slow activation, reduced inactivation.<br>w/ E1: activation $\Delta V_{1/2}$ left shift, fast activation, co-assembly with E1 decreased.	(25)
**V307L**	GTG>CTG/TTG	Pore helix	SQTS	pathogenic	w/ E1: activation $\Delta V_{1/2}$ left shift and instantaneous activation, fast activation, slow deactivation.	(14,18)
**R670K**	AGG>AAG	C-terminus	AF	not provided	w/o and w/ E1: I_{max} increased.	(27,30)

LQTS: long QT syndrome   SQTS: short QT syndrome   AF: atrial fibrillation   E: KCNE

Table S2. Trafficking results of KCNQ1 mutants in HEK293 and CHO cells

Mutation	Cell surface expression (%WT)	Total expression (%WT)	Trafficking efficiency (%WT)
**HEK293 cells**			
**WT**	100 ± 0	100 ± 0	100 ± 0
**F166M**	67 ± 2	57 ± 1	118 ± 4
**R231C**	501 ± 16	167 ± 6	302 ± 8
**R231M**	328 ± 8	136 ± 2	241 ± 3
**R231F**	151 ± 17	107 ± 5	140 ± 10
**F166M-R231F**	250 ± 8	117 ± 2	213 ± 4
**V129A**	91 ± 4	74 ± 1	123 ± 4
**V129A-R231C**	607 ± 50	201 ± 20	304 ± 15
**CHO cells**			
**WT**	100 ± 0	100 ± 0	100 ± 0
**F166M**	69 ± 8	64 ± 4	107 ± 7
**R231C**	472 ± 32	191 ± 14	267 ± 38
**R231F**	201 ± 13	146 ± 5	138 ± 12
**F166M-R231F**	216 ± 21	158 ± 12	137 ± 11
**V129A**	101 ± 7	85 ± 3	118 ± 7
**V129A-R231C**	364 ± 36	209 ± 5	174 ± 16

Data are expressed as mean ± SEM of at least four independent biological replicates.
